# The Role of Advanced Cardiac Imaging in Monitoring Cardiovascular Complications in Patients with Extracardiac Tumors: A Descriptive Review

**DOI:** 10.3390/jcdd12010009

**Published:** 2024-12-29

**Authors:** Annamaria Tavernese, Valeria Cammalleri, Rocco Mollace, Giorgio Antonelli, Mariagrazia Piscione, Nino Cocco, Myriam Carpenito, Carmelo Dominici, Massimo Federici, Gian Paolo Ussia

**Affiliations:** 1 Department of Medicine and Surgery, Università Campus Bio-Medico di Roma, Via Alvaro del Portillo 21, 00128 Roma, Italy; 2 Operative Research Unit of Emodinamica, Fondazione Policlinico Universitario Campus Bio-Medico, Via Alvaro del Portillo 200, 00128 Roma, Italy; 3 Department of Experimental Medicine, Università degli Studi di Roma Tor Vergata, Via Montpellier 1, 00133 Rome, Italy; 4 Cardiology Unit, Humanitas Gavazzeni, 24125 Bergamo, Italy; 5 Operative Research Unit of Cardiac Surgery, Fondazione Policlinico Universitario Campus Bio-Medico, Via Alvaro del Portillo 200, 00128 Roma, Italy

**Keywords:** cardiotoxicity, echocardiography, cardiac computed tomography (CT), cardiac magnetic resonance (CMR), radiation-induced heart disease, extracardiac tumors, cancer therapy-related cardiac dysfunction (CTRCD), global longitudinal strain (GLS), cardiovascular risk assessment in cancer, myocardial tissue characterization

## Abstract

Cardiac involvement in cancer is increasingly important in the diagnosis and follow-up of patients. A thorough cardiovascular evaluation using multimodal imaging is crucial to assess any direct cardiac involvement from oncological disease progression and to determine the cardiovascular risk of patients undergoing oncological therapies. Early detection of cardiac dysfunction, particularly due to cardiotoxicity from chemotherapy or radiotherapy, is essential to establish the disease’s overall prognostic impact. Comprehensive cardiovascular imaging should be integral to the clinical management of cancer patients. Echocardiography remains highly effective for assessing cardiac function, including systolic performance and ventricular filling pressures, with speckle-tracking echocardiography offering early insights into chemotoxicity-related myocardial damage. Cardiac computed tomography (CT) provides precise anatomical detail, especially for cardiac involvement due to metastasis or adjacent mediastinal or lung tumors. Coronary assessment is also important for initial risk stratification and monitoring potential coronary artery disease progression after radiotherapy or chemotherapeutic treatment. Finally, cardiac magnetic resonance (CMR) is the gold standard for myocardial tissue characterization, aiding in the differential diagnosis of cardiac masses. CMR’s mapping techniques allow for early detection of myocardial inflammation caused by cardiotoxicity. This review explores the applicability of echocardiography, cardiac CT, and CMR in cancer patients with extracardiac tumors.

## 1. Introduction

Extracardiac tumors encompass a wide range of malignancies that originate outside the heart but can have significant effects on the cardiovascular system. These malignancies may affect heart function either by metastasizing to cardiac structures or through the cardiotoxicity induced by cancer treatments [1]. Advances in oncologic therapies have notably improved survival rates; however, they have also underscored the importance of managing the cardiovascular side effects that frequently arise during and after treatment [2]. In fact, cardiovascular disease has become one of the leading causes of morbidity and mortality among cancer survivors, making regular assessment of heart health a priority during oncologic care [3,4,5].

A growing concern is the cardiac impact of cancer therapies, particularly chemotherapy, radiotherapy, and newer immunotherapies. Agents such as anthracyclines and HER2-targeted therapies are well-known for their potential to cause cardiomyopathy, which can progress to heart failure if not recognized and managed early [1]. Similarly, radiation therapy—especially when the mediastinal area is involved—has been linked to the development of coronary artery disease, valvular dysfunction, and pericarditis. These effects may take years to manifest, necessitating long-term follow-up [2,6].

Radiation-induced damage to coronary arteries and heart valves leads to accelerated atherosclerosis and calcific valvular disease, conditions best identified through advanced imaging modalities such as cardiac computed tomography (CT) [7]. Immune checkpoint inhibitors, a class of immunotherapies, have also been associated with myocarditis and other serious cardiac events. Although rare, myocarditis can be life-threatening if not detected early and has been increasingly documented with agents like nivolumab and pembrolizumab [8,9]. Cardiovascular complications related to these therapies, although infrequent, require vigilant cardiac imaging to ensure timely detection and intervention [10].

Given the rising importance of cardiovascular care in oncology, advanced cardiac imaging has become indispensable in detecting, monitoring, and managing heart-related complications. Modalities such as echocardiography, cardiac magnetic resonance (CMR), and CT are crucial for providing comprehensive assessments of both functional and structural aspects of cardiac health in cancer patients [3]. These imaging techniques not only facilitate the diagnosis of cardiotoxicity but also help in monitoring the progression of the disease and evaluating responses to cardioprotective therapies [11]. The use of advanced imaging techniques like strain echocardiography, T1/T2 mapping in CMR, and coronary artery calcium (CAC) scoring in CT enhances the ability to detect subclinical cardiac dysfunction before it leads to irreversible damage [12,13,14].

This review aims to provide an updated overview of the role of advanced cardiac imaging modalities in cancer patients, focusing on echocardiography, cardiac CT, and CMR. By discussing their applications, strengths, and limitations, we highlight the critical role of a multidisciplinary approach in optimizing outcomes for patients who may be at risk of cardiovascular complications from cancer treatment.

## 2. Cardiac Complications of Cancer Therapy

While cancer therapies have significantly improved survival rates, they are also associated with an increased risk of cardiovascular complications. It is essential to carefully manage and monitor these risks to balance oncologic efficacy with heart health [1,2,4].

### 2.1. Chemotherapy-Induced Cardiotoxicity

Chemotherapeutic agents, particularly anthracyclines like doxorubicin, are known to cause dose-dependent cardiotoxicity, leading to various forms of myocardial injury. This damage may evolve into symptomatic heart failure, with mechanisms rooted in oxidative stress, mitochondrial dysfunction, and cardiomyocyte apoptosis, followed by fibrosis that can occur even years after treatment completion [15,16]. Early recognition and intervention are crucial to prevent irreversible damage to the myocardium [17].

HER2-targeted therapies such as trastuzumab pose additional cardiac risks, especially when combined with anthracyclines. These agents inhibit the HER2 pathway, which plays a key role in maintaining cardiac cell survival, particularly under stress. Consequently, trastuzumab-related cardiotoxicity is often more severe in patients with prior anthracycline exposure [18]. Unlike anthracycline damage, trastuzumab-induced cardiotoxicity is often reversible upon cessation, but underlying heart conditions can amplify the risk of long-term cardiac dysfunction.

Additionally, cyclophosphamide and ifosfamide are known to directly cause endothelial damage through toxic metabolite leakage, leading to injury of both cardiomyocytes and interstitial tissues [19].

Furthermore, plant alkaloids like paclitaxel and docetaxel increase the risk of coronary artery disease (CAD) by promoting endothelial damage. Their mechanism of action involves the inhibition of cell proliferation, resulting in abnormal microtubule formation, which compromises the integrity of the endothelial layer. This damage facilitates the migration of low-density lipoproteins (LDL) and leukocytes into the subintimal space, promoting atheroma formation [20].

### 2.2. Radiotherapy-Associated Cardiovascular Disease

Radiation therapy, especially for mediastinal tumors, can lead to long-term cardiovascular complications such as CAD, valvular disease, and pericarditis [6]. The primary pathophysiological mechanism involves endothelial injury, which triggers chronic inflammation, accelerates atherosclerosis, and leads to calcification in the coronary arteries and cardiac valves [7]. These changes often manifest years after treatment, underlining the need for lifelong cardiovascular monitoring in cancer survivors [21].

Valvular heart disease is a particularly common late effect of radiotherapy, often requiring valve replacement due to significant calcification [22]. In particular, young patients who have received mediastinal radiation are at high risk for developing aortic or mitral valve disease decades after treatment [23].

### 2.3. Immunotherapy and Cardiotoxicity

Immune checkpoint inhibitors (ICIs) like nivolumab and pembrolizumab, which have revolutionized cancer treatment, are associated with myocarditis, a rare but potentially fatal side effect. The incidence of myocarditis with ICIs is estimated at 0.04% to 1.14%, with delayed presentations occurring up to one year after treatment initiation [8]. These cases can range from high-grade to low-grade presentations [24]. Given the high mortality associated with this condition, regular cardiac monitoring remains crucial [10].

In addition to myocarditis, ICIs have been linked to increased risks of acute coronary syndromes (ACS) and arrhythmias like atrial fibrillation, likely due to systemic inflammation and immune activation, which destabilizes atherosclerotic plaques and exacerbates coronary artery disease [25].

## 3. Echocardiography

Echocardiography remains the cornerstone of cardiac evaluation in cancer patients due to its accessibility, affordability, and safety profile. The European Society of Cardiology (ESC) guidelines emphasize the central role of transthoracic echocardiography (TTE) for the baseline assessment of cardiac function and structure before initiating chemotherapy [1]. Its importance lies in its ability to detect chemotherapy-induced cardiac dysfunction, particularly in patients treated with anthracyclines, which are known to cause cardiomyopathy. According to current guidelines, echocardiography should be used to evaluate cardiac function throughout various stages of cancer treatment, from the presentation of heart failure symptoms to monitoring during therapy and follow-up, extending up to one-year post-treatment [26]. Furthermore, several expert panels have issued recommendations on specific echocardiographic protocols, including the use of strain imaging to monitor cardiotoxicity induced by anthracyclines [3].

Two-dimensional (2D) echocardiography remains the standard technique for assessing left ventricular ejection fraction (LVEF), a key marker for detecting chemotherapy-induced cardiotoxicity [3]. Cardiotoxicity induced by oncology therapies is typically identified by a decrease in left ventricular ejection fraction to less than 40%, or a reduction of 10% or more to a range between 40% and 49% [26]. However, relying solely on LVEF may not reveal subclinical cardiac dysfunction, as it typically occurs later in the disease process. This limitation underscores the growing role of advanced techniques like strain imaging and three-dimensional (3D) echocardiography in early detection [27].

**Figure 1:**
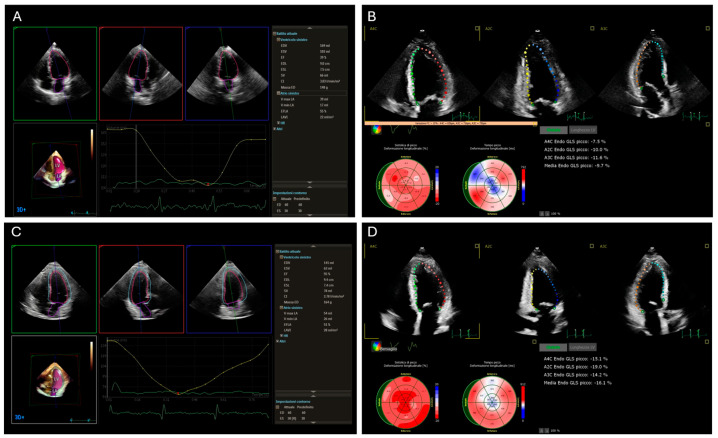
Global longitudinal strain (GLS) analysis and three-dimensional (3D) left ventricle ejection fraction (LVEF) of a breast cancer patient undergoing trastuzumab therapy. Significant reduction in 3D LVEF and GLS after 6 months of treatment (Panel **A** + Panel **B**). Improvement of 3D LVEF and GLS after 4 months post-therapy cessation and initiation of cardioprotective treatment (Panel **C** + Panel **D**).

Global longitudinal strain (GLS) by speckle-tracking echocardiography (STE) is highly sensitive in detecting early myocardial dysfunction, even before a decline in LVEF becomes apparent. GLS is particularly useful in patients receiving cardiotoxic chemotherapeutic agents such as anthracyclines or HER2-targeted therapies [28,29] (Figure 1).

Early detection through GLS allows for timely intervention with cardioprotective therapies, which can improve patient outcomes by preventing progression to symptomatic heart failure [30].

While GLS is well-established in cardio-oncology, recent studies have shed light on the role of left atrial (LA) strain, which is also gaining importance in cancer patients. LA strain, particularly the LA reservoir strain, is an early and sensitive marker of myocardial fibrosis and elevated left ventricular filling pressures. It has been introduced as a standard parameter in diagnosing diastolic dysfunction in heart failure with preserved ejection fraction (HFpEF) [31]. Additionally, LA strain has a prognostic role in heart failure patients, with studies showing that reduced LA contractile function correlates with worse outcomes in heart failure with reduced ejection fraction (HFrEF) [32].

A multicenter study by Di Lisi et al. [33] emphasized the utility of LA strain assessment using STE in breast cancer patients treated with chemotherapy. The study found that atrial function changes significantly during chemotherapy, and a greater impairment of LA strain was observed in patients who developed asymptomatic mild cancer therapy-related cardiac dysfunction (CTRCD). A reduction in peak atrial longitudinal strain (PALS) by 20.8% was identified as a cutoff for detecting patients at higher risk of cardiotoxicity. This suggests that assessing LA strain alongside GLS could provide a more comprehensive and early detection of subclinical cardiotoxicity, helping clinicians intervene earlier to prevent progression to more severe cardiac dysfunction.

However, despite its advantages, GLS is not without limitations. Its accuracy can be affected by factors such as image quality, acquisition techniques, and the use of vendor-specific algorithms for data processing [34]. Additionally, GLS measurements are influenced by changes in chamber geometry and left ventricular remodeling, as well as by hemodynamic variables such as heart rate and loading conditions [34]. These factors necessitate careful interpretation of GLS findings, particularly in complex cardio-oncology cases.

To overcome some of the limitations of GLS, myocardial work (MW) indices, derived from left ventricular pressure-strain loops, offer a more comprehensive assessment by incorporating blood pressure data. This helps account for loading conditions that can affect GLS readings, making MW particularly valuable in cardio-oncology, where cancer treatments often cause blood pressure fluctuations that impact myocardial function. Indices like the global work index (GWI) and global constructive work (GCW) provide a more accurate measure of myocardial performance in these patients [35,36,37].

Several studies have explored the utility of MW in cancer patients [38]. Guan et al. found that while GLS was sensitive to cardiac dysfunction, changes in MW indices were more pronounced after the sixth cycle of treatment, suggesting that MW may detect early myocardial dysfunction more sensitively [39]. However, Calvillo-Argüelles et al. reported that MW did not offer substantial advantages over GLS in predicting CTRCD in patients treated with anthracycline and trastuzumab, especially when clinical risk factors were considered [40]. Kosmala et al. demonstrated that MW indices could differentiate between chemotherapy-induced myocardial changes and those caused by afterload. Patients with CTRCD but no blood pressure rise showed larger reductions in GWI and GCW, suggesting that MW can help distinguish between myocardial dysfunction and afterload-related changes [41]. Similarly, Vaz Ferreira et al. observed significant MW impairments in breast cancer patients treated with anthracycline or anti-HER therapies, with recovery to baseline levels occurring 12–14 months post-treatment. Patients with CTRCD showed a more significant decline in these indices early on [42]. Although MW indices appear to offer a more sensitive and timely evaluation of myocardial function in cardio-oncology, further research is needed to fully understand their clinical significance, particularly regarding long-term outcomes such as heart failure risk and treatment strategies.

Right ventricular (RV) function is also significantly affected in patients treated with anthracyclines. Recent studies have demonstrated that RV function, evaluated through STE and 3D ejection fraction (EF), tends to decline over time, peaking around six months post-treatment and stabilizing at twelve months [43]. The synchronous impairment of both RV and LV suggests that cardiotoxicity may be a global process affecting the entire heart, not limited to one ventricle. As such, comprehensive biventricular assessment is critical in cancer patients [44].

Advanced echocardiographic techniques like STE and 3D echocardiography are superior in detecting subclinical RV damage compared to conventional methods. Notably, the detection of subclinical ventricular dysfunction using these techniques does not always correlate with a reduction in traditional parameters or the onset of symptomatic CTRCD. This underscores the value of advanced imaging in early detection [43].

Stress echocardiography has emerged as a valuable tool in the cardio-oncology setting, particularly for the evaluation of ischemic heart disease. It is often employed to assess CAD in patients with intermediate pretest probability or those undergoing treatments associated with an increased risk of ischemia, such as 5-fluorouracil, bevacizumab, sorafenib, and sunitinib [3,45]. Additionally, stress echocardiography is useful for evaluating subclinical left ventricular dysfunction and assessing contractile reserve, offering valuable insights into patients at risk of late-onset cardiac dysfunction [46].

Recent studies by Novo et al. demonstrated that assessing left ventricular contractile reserve (LVCR) during stress echocardiography can identify patients more likely to develop cardiac dysfunction following high-dose chemotherapy (HDC), even when LVEF and diastolic function appear normal. Early detection through this method allows for timely intervention, improving long-term outcomes in at-risk patients [47].

Radiotherapy, particularly for mediastinal tumors, is known to cause various cardiovascular complications, including diastolic dysfunction and valvular heart disease. These complications arise from radiation-induced endothelial damage, which leads to chronic inflammation, fibrosis, and calcification of cardiac structures [6,48,49]. Echocardiography, with techniques like tissue Doppler and strain imaging, is particularly effective in assessing diastolic dysfunction caused by radiation therapy. This modality allows for a detailed evaluation of the degree of diastolic impairment and can help in the early detection of radiation-induced heart disease [50].

Additionally, contrast-enhanced echocardiography (CEE) is a significant tool in the differentiation of cardiac masses, helping distinguish between thrombi, metastases, and benign tumors. In oncology patients, where intracardiac masses may be the result of metastasis, tumor extension, or thrombus formation, CEE plays a crucial role in accurate diagnosis. The addition of contrast agents enhances the visualization of endocardial borders and helps delineate the perfusion characteristics of intracardiac masses, improving diagnostic accuracy [51]. For example, a thrombus typically appears as a non-perfused mass, while a tumor or metastasis often shows vascular enhancement due to its blood supply. This distinction is critical for determining the appropriate management strategy, whether it be anticoagulation, surgical removal, or adjustments to cancer therapy [52].

In conclusion, echocardiography remains an essential imaging tool in the management of cancer patients. Techniques such as GLS, myocardial work indices, and advanced modalities like 3D echocardiography enable clinicians to detect subclinical cardiac dysfunction and guide appropriate interventions. Despite its wide availability and non-invasive nature, echocardiography does have certain limitations. Its diagnostic accuracy and reliability can be affected by operator dependency, which may lead to variability in measurements. Additionally, factors such as poor acoustic windows—often due to obesity, lung interference, or anatomical variations—can reduce image quality, posing challenges in some patients. Variability in inter- and intra-observer measurements further underscores the importance of training and standardization in clinical practice.

Nevertheless, these challenges are outweighed by its accessibility and versatility, making echocardiography invaluable for monitoring and managing cardiotoxicity in cancer therapy [26].

## 4. Cardiac Magnetic Resonance (CMR) Imaging

Cardiac magnetic resonance imaging (CMR) has become a critical imaging modality in the assessment and monitoring of cardiotoxicity in oncology patients. Its comprehensive ability to assess both structural and functional parameters of the myocardium with high spatial resolution makes CMR one of the most powerful tools for evaluating cancer therapy-related cardiac dysfunction [53]. Unlike echocardiography, which is often limited by acoustic windows and operator variability, CMR provides precise and reproducible measurements of LVEF, as well as other critical parameters such as myocardial strain, myocardial tissue characteristics, and scar burden through late gadolinium enhancement (LGE) [54].

**Figure 2:**
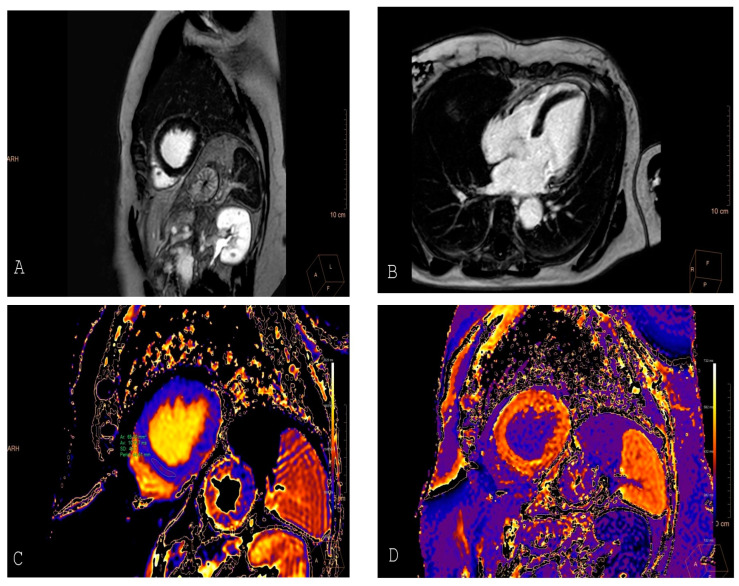
A 47-year-old patient undergoing anthracycline therapy for the treatment of breast cancer underwent a cardiac MRI following the detection of a mild reduction in left ventricular ejection fraction on echocardiography: post-contrast sequences do not reveal areas of myocardial late gadolinium enhancement (Panel **A** and Panel **B**). Native T1 mapping, measured by placing a ROI in the mid interventricular septum, is slightly increased (Panel **C**). The ECV, obtained through post-contrast T1 mapping sequences, is diffusely elevated (Panel **D**). These findings are indicative of diffuse interstitial inflammation due to chemotherapy-induced toxicity, supporting the prognostic role of T1 mapping independently.

The use of CMR is especially important in cardio-oncology due to its ability to detect subtle myocardial changes before significant clinical symptoms develop. CMR’s sensitivity in identifying myocardial edema, fibrosis, and scar formation is instrumental in monitoring patients undergoing potentially cardiotoxic cancer therapies, such as anthracyclines and HER2-targeted agents [15,54]. T1-weighted imaging represents the intrinsic composition of both intracellular and extracellular tissue. Each pixel is given an intensity value, which is unitless and referenced to a “region of interest” within the same image. T1 is increased by water, edema, and inflammation. In T1 mapping images, each colored pixel corresponds to a quantified, tissue-specific T1 value for the respective voxel, allowing for comparison across different images. Contrast-enhanced T1 mapping is primarily used to calculate the extracellular volume (ECV) fraction, often in conjunction with native T1 mapping [55,56] (Figure 2). Myocardial fibrosis, a hallmark of cardiotoxicity, is detectable through T1 mapping and ECV quantification. These CMR-derived techniques can identify diffuse myocardial changes even in the absence of LGE, which is typically used to detect focal fibrosis [57]. T1 and T2 mapping allows for the assessment of both extracellular matrix expansion and myocardial edema, which are early signs of injury, often preceding irreversible damage [58]. Furthermore, recent studies have emphasized the role of T1 and T2 mapping as valuable tools for assessing myocardial changes in patients undergoing anthracycline or trastuzumab therapy. A study by Haslbauer et al. highlighted the usefulness of T1 and T2 mapping in detecting early myocardial abnormalities that correlate with cardiac dysfunction in cancer patients, even before LVEF drops below the normal limit [14]. Building upon these findings, the prognostic role of CMR in chemotherapy-induced transient left ventricular dysfunction is particularly noteworthy. The advanced capabilities of CMR, such as T1 and T2 mapping, allow for the early detection of myocardial edema and fibrosis, which are often subclinical yet predictive of adverse outcomes. These imaging techniques enable clinicians to identify myocardial injury at a reversible stage while also providing crucial information for implementing cardioprotective strategies [59]. This positions CMR not only as a diagnostic tool but also as a prognostic one, offering valuable insights to guide early interventions and improve long-term cardiovascular outcomes.

Notably, recent advancements in myocardial T1ρ mapping have been applied to follow-up on patients with hypertrophic cardiomyopathy, which can also serve as a potential tool in monitoring myocardial changes in oncology patients. T1ρ imaging has the potential to directly detect interstitial fibrosis and other myocardial disease processes by targeting large molecules, rather than indirectly detecting changes through their influence on water, as seen with T1 and T2 relaxation. These advanced mapping techniques provide high sensitivity in detecting alterations in myocardial relaxation properties, a key feature in the early detection of cardiotoxicity [60]. Bustin et al. also emphasized the value of T1ρ mapping in providing more granular information about myocardial health, especially in the context of treatment-related changes [61].

The application of LGE is also of paramount importance in cardio-oncology, particularly in differentiating between reversible and irreversible myocardial injury. LGE helps in distinguishing between fibrosis caused by ischemic versus non-ischemic etiologies, which is crucial in patients undergoing cancer therapies known to induce ischemic injury, such as certain chemotherapies or targeted agents. For instance, LGE patterns seen in patients treated with anthracyclines or radiotherapy often indicate non-ischemic fibrosis, while HER2-targeted agents may cause more diffuse myocardial changes [62,63]. In radiation-associated cardiotoxicity, LGE often reveals fibrotic changes in both the myocardium and pericardium, which may be indicative of radiation-induced injury to cardiac structures [64].

In addition to LGE, myocardial strain assessment by CMR tagging or feature tracking is gaining traction for evaluating cardiotoxicity. This approach allows for detailed analysis of regional and global myocardial deformation, providing insights into both systolic and diastolic function. CMR feature tracking has shown promising results in detecting subtle myocardial dysfunction in patients undergoing cancer therapy, particularly when traditional parameters such as LVEF remain within the normal range [65]. This non-invasive, highly sensitive modality allows for the detection of early myocardial contractile dysfunction, which can be crucial for initiating timely cardioprotective measures.

One of the emerging applications of CMR in cardio-oncology is in the evaluation of cardiotoxicity in patients receiving immune checkpoint inhibitors that have been associated with myocarditis, where CMR is the gold standard for diagnosing myocarditis due to its unparalleled ability to characterize myocardial tissue. T2 mapping for myocardial edema and LGE for fibrosis can confirm the diagnosis, differentiate between acute and chronic phases, and evaluate the severity of inflammation [66]. Furthermore, in myocarditis related to immune checkpoint inhibitors, higher T1 native values, detected by the T1 mapping technique, showed evidence of greater myocardial injury and were independently associated with MACE [67].

Additionally, CMR is valuable for its ability to assess right ventricular function, which can be particularly affected by cancer therapies, especially those involving radiation to the chest or those that impact the pulmonary vasculature. Right ventricular dysfunction is a significant predictor of outcomes in patients with cancer-related cardiotoxicity, and CMR provides a comprehensive evaluation of RV volumes, ejection fraction, and myocardial tissue characterization, which are superior to echocardiographic assessments [14].

Another significant aspect of CMR in cardio-oncology is its role in monitoring cardiotoxicity associated with proteasome inhibitors. CMR helps in detecting early myocardial dysfunction, even when patients remain asymptomatic, thereby guiding timely interventions [68]. This is critical as the cardiovascular side effects of these drugs can be severe, necessitating the use of CMR as part of the routine follow-up in high-risk patients.

Finally, CMR is instrumental in assessing diastolic dysfunction, which is often an early manifestation of cardiotoxicity. By utilizing techniques like T1 and T2 mapping, CMR can detect changes in the myocardium that indicate increased stiffness or impaired relaxation, even before clinical symptoms manifest, or traditional diastolic parameters are affected. This comprehensive ability makes CMR an invaluable tool for identifying early cardiotoxic effects and guiding treatment decisions [14,58].

In conclusion, CMR provides a comprehensive evaluation of cardiac function and structure, offering insights beyond those available from other imaging modalities. It is particularly valuable in detecting early cardiotoxic changes through techniques such as T1/T2 mapping, LGE, and strain analysis. Its role in differentiating reversible from irreversible myocardial injury, assessing both left and right ventricular function, and evaluating cardiotoxicity related to various cancer therapies makes it a cornerstone of cardio-oncology imaging. By providing a detailed understanding of myocardial tissue characteristics and early dysfunction, CMR is pivotal in the timely identification and management of patients at risk of CTRCD, thus contributing to better overall outcomes in cancer patients [11,58].

CMR is a valuable imaging tool in cardio-oncology, offering high spatial resolution and detailed tissue characterization. However, its widespread use faces several challenges. Limited availability and high costs restrict CMR mainly to specialized centers, making it less accessible for routine monitoring [14,69]. Patient-related factors, such as non-MRI-compatible implants and claustrophobia, further limit its applicability. The use of gadolinium contrast carries risks, particularly in patients with renal impairment, due to the possibility of nephrogenic systemic fibrosis [70].

The long scan duration of CMR can also lead to reduced patient compliance resulting in poor image quality due to motion artifacts [71]. Monitoring cardiac function longitudinally also presents challenges, as repeated CMR is less practical due to logistical and financial barriers, compounded by variability in imaging protocols [72].

Overall, these limitations highlight the need for a multimodality imaging approach in cardio-oncology, using CMR in conjunction with other techniques like echocardiography and cardiac CT to provide a comprehensive cardiac assessment while managing patient-specific challenges and resource constraints.

## 5. Cardiac Computed Tomography (CT)

Cardiac computed tomography (CT) has become an invaluable tool in cardio-oncology, especially for the evaluation of CAD in cancer patients and survivors, who are at an elevated risk compared to non-cancer patients due to prior cancer treatments, including chemotherapy and radiation therapy. Its application is critical in assessing cardiovascular complications that often arise following thoracic radiation or cardiotoxic chemotherapy, providing comprehensive anatomical visualization and risk assessment [73].

**Figure 3:**
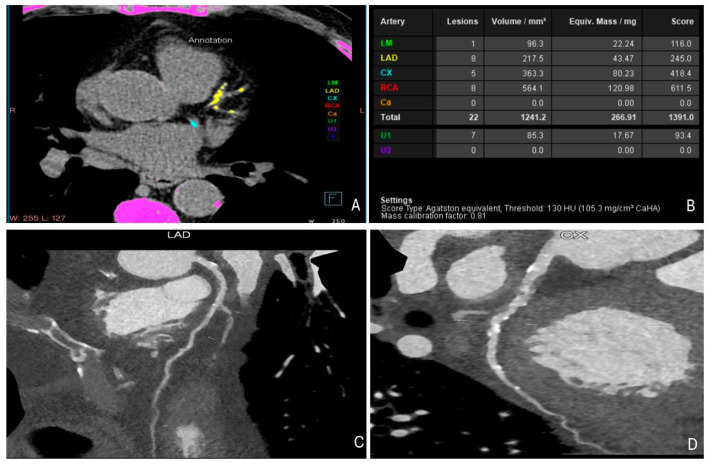
A 62-year-old patient with a history of prior exposure to radiotherapy for the treatment of Hodgkin’s lymphoma underwent a computed tomography scan for atypical angina. High pitch spiral (FLASH) coronary artery calcium (CAC) imaging shows diffuse calcifications of the coronary arteries; a CT attenuation threshold of 130 Hounsfield units (HU) is used for the detection of calcium (Panel **A**); a CAC score of 1391 Ag was obtained using an equivalent scoring method to the Agatston score (Panel **B**); The subsequent coronary CT angiography reveals diffuse coronary atheromatous with significant stenosis of the circumflex artery (Panel **C** and Panel **D**).

One of the most valuable roles of cardiac CT in cardio-oncology is the assessment of coronary artery calcium (CAC) scoring (Figure 3). CAC scoring helps to evaluate the atherosclerotic burden and assess the future risk of cardiovascular events, which is particularly important in cancer survivors, especially those treated with radiation. For instance, breast cancer survivors exposed to radiation have a higher prevalence of coronary artery calcification, which serves as an important predictor of CAD development [74]. Studies, such as those by Chang et al., have further highlighted the increased coronary calcium observed in breast cancer survivors after radiation therapy, underlining the importance of cardiac CT in this patient group [75].

There is strong evidence supporting the prognostic value and utility of CAC score assessment in non-gated CT scans. Incorporating baseline CAC screening in cancer patients using non-gated CT scans, such as staging chest CTs or the CT component of PET scans, can aid in risk stratification and early diagnosis of atherosclerotic cardiovascular disease (ASCVD) [76]. This modality enables clinicians to stratify cardiovascular risk effectively, providing insight that may prompt the initiation of preventative measures, such as the use of cardioprotective agents, tailored to the individual risk profile of each patient [77]. The BRAGATSTON clinical trial investigated whether automated quantification of CAC on radiation therapy (RT) planning chest CTs could be useful for predicting cardiovascular disease (CVD) risk. The researchers concluded that automated CAC scoring could serve as a quick and cost-effective method for identifying breast cancer patients who are at higher risk of developing CVD [78].

The Society of Cardiovascular Computed Tomography (SCCT) has developed a straightforward system called the CAC-Data Reporting System (CAC-DRS). This system combines quantitative assessments given by Agatson score systems, with qualitative visual assessments of calcified coronary plaque burden to help estimate cardiovascular disease (CVD) risk [79].

CAC scores can easily be derived from CT scans performed for lung cancer screening, offering valuable information for CVD risk stratification. These scores are also predictive of CVD events, supporting the complementary use of cancer screening and surveillance methods to enhance CVD risk prediction [80].

Cardiac CT also plays an important role in evaluating radiation-induced coronary artery disease. It is evident that in patients who have undergone RT, atherosclerotic cardiovascular disease tends to develop in the coronary arteries exposed to the radiation. Furthermore, the risk of coronary events is directly related to the radiation dose received. Patients who have undergone mediastinal radiation for cancers such as Hodgkin lymphoma are at a significant risk for CAD. This increased risk has been documented through studies like those conducted by van Nimwegen et al., who demonstrated a radiation dose-response relationship for CAD in cancer survivors [81]. Due to the high incidence of atherosclerosis following radiation exposure, many experts suggest preferring a functional evaluation rather than an anatomical test [26]. However, coronary CTA is a useful alternative for patients with suspected CAD, offering a high negative predictive value for ruling out obstructive CAD. Unlike functional stress tests, coronary CTA has the added benefit of detecting atherosclerotic plaque that might otherwise go unnoticed, in order to allow an early stabilization treatment of the atherosclerotic plaque [82]. Furthermore, in the last decade, new technological improvements such as FFRct and CT perfusion have made it possible to combine anatomical and functional evaluation [83].

Additionally, coronary CT angiography (CTCA) has become an important diagnostic modality for identifying coronary stenosis in cancer survivors, particularly those treated with high-risk chemotherapies [84]. Vascular toxicities have become the second most common category of adverse effects linked to anticancer therapies. Arterial toxicities can manifest as acute vasospasm, acute thrombosis, or accelerated atherosclerosis [85]. Particularly in patients treated with platinum drugs, 5-fluorouracil and cyclophosphamide, which have a direct toxic effect by promoting a prothrombotic state, CCTA could play a role in excluding CAD associated with the symptoms, but also in defining the risk before starting treatment [86]. Furthermore, CTCA is recognized as a non-invasive alternative to coronary angiography for assessing coronary artery disease in patients with newly diagnosed heart failure and new onset left ventricular systolic dysfunction. It helps differentiate between ischemic and non-ischemic causes, particularly in patients with low to intermediate risk who start drugs known to have direct myocardium toxicity [87].

Pericardial disease is a frequent and serious complication in cancer patients, often manifesting as acute pericarditis, pericardial effusion, or constrictive pericarditis [88]. Therapeutic radiation can have detrimental effects on the pericardium, with these issues arising acutely but more frequently manifesting years or even decades after treatment, especially at contemporary radiation doses. This is particularly common in patients treated for mediastinal tumors or breast cancer [89]. Cardiac CT is highly sensitive in detecting pericardial calcification, a key indicator of constrictive pericarditis and it can be useful in identifying signs of ventricular interdependence, such as septal flattening, retrograde contrast flow into the inferior vena cava, and bi-atrial enlargement, with the help of multiphase ECG-gated imaging [90]. Moreover, cardiac CT can detect acute pericarditis by showing iodinated contrast enhancement of the visceral and parietal pericardial surfaces and is able to give us information regarding the nature of the pericardial effusion [91].

In recent years, cardiac CT has also been used to evaluate the extent of myocardial fibrosis in cancer patients. Quantifying ECV is one such application where cardiac CT has demonstrated promise, similar to its role in cardiac MRI, by detecting myocardial changes that occur in response to cardiotoxic therapies [92]. Elevated ECV values indicate the presence of fibrosis, which is often associated with poor cardiovascular outcomes, emphasizing the role of cardiac CT in comprehensive cancer care.

In conclusion, cardiac CT serves as a versatile tool in the assessment of cardiovascular health in cancer patients. By utilizing techniques such as CAC scoring and CTCA, cardiac CT helps in identifying both calcific and non-calcific atherosclerosis, assessing CAD risk, and managing long-term cardiovascular outcomes in cancer survivors. While careful consideration must be given to the risks associated with radiation exposure and nephrotoxicity from contrast agents, other limitations include the potential for artifacts, particularly in patients with heavy coronary calcifications, and the requirement for patient preparation, such as heart rate control, which may not be suitable for all individuals. Additionally, cardiac CT is less accessible in resource-limited settings due to its high cost and the need for specialized equipment and expertise.

Despite these challenges, the detailed anatomical insights provided by cardiac CT make it an essential modality for cardiac surveillance and management in patients undergoing cancer therapy. This comprehensive approach aids in the early detection of coronary and myocardial abnormalities, enabling timely intervention and reducing cardiovascular morbidity in a high-risk population [93,94,95].

## 6. Future Perspectives

Innovations in cardiac imaging are rapidly advancing, promising to significantly enhance the diagnosis, monitoring, and management of cardiotoxicity in cancer patients. Emerging technologies such as four-dimensional flow cardiac magnetic resonance imaging (4D flow CMR) can be applied in assessing left ventricular diastolic function. This advanced imaging technique is useful in evaluating blood flow dynamics and left ventricular function, which may help in detecting early stages of diastolic dysfunction. By capturing 3D velocity data over time, 4D flow CMR allows a detailed analysis of complex hemodynamics, offering more comprehensive insights than traditional methods. This real-time volumetric imaging provides insights into subtle changes in heart mechanics, offering early detection of treatment-related dysfunctions [96]. Additionally, artificial intelligence (AI) is being integrated into cardiac imaging for more accurate image interpretation, reducing human error, and speeding up the diagnostic process. AI algorithms can enhance pattern recognition in imaging datasets, helping predict outcomes and guide interventions in cardio-oncology [97].

Moreover, molecular imaging technologies, such as positron emission tomography (PET)/CT, are paving the way for early detection of molecular changes in myocardial tissue before structural abnormalities become apparent. These advancements have the potential to improve risk stratification and personalized treatment in oncology patients, leading to better cardiac outcomes [98].

Another promising innovation is photon counting computed tomography (PCCT), which represents a significant advancement with potential implications for the evaluation of epicardial coronary arteries in oncology patients. PCCT utilizes photon-counting detectors to achieve ultra-high-resolution imaging and enhanced material differentiation compared to conventional CT systems. This capability is particularly relevant in cardio-oncology, where precise visualization of coronary anatomy and plaque characterization can facilitate early detection and management of coronary artery disease. By minimizing calcium blooming artifacts, PCCT improves the accuracy of stenosis assessment, even in patients with extensive calcifications—a common challenge in those who have undergone radiotherapy. Furthermore, the ability of PCCT to operate at reduced radiation doses makes it a safer option for long-term surveillance in cancer survivors, while its spectral imaging capabilities allow for better differentiation of tissue types, enhancing diagnostic precision.

Although the direct application of PCCT in cardio-oncology is still at an early stage, future research exploring its potential could position it as a pivotal imaging modality in this field. Studies focusing on its diagnostic accuracy, cost-effectiveness, and integration with existing multimodality imaging protocols are essential to establish its role in routine clinical practice [99,100,101].

Personalized medicine is becoming an integral part of oncologic care, where cardiac imaging plays a pivotal role in tailoring treatments. By utilizing imaging tools, clinicians can adapt therapies and follow-up protocols to each patient’s specific cardiovascular profile, balancing cancer treatment efficacy with the minimization of cardiac risks. This approach allows for the identification of high-risk patients early in their treatment course and the application of preventive cardioprotective therapies [102].

The potential for combining different imaging modalities, such as CMR and PET, offers a more comprehensive evaluation of both structural and metabolic changes in the heart. For example, combining CMR’s detailed assessment of myocardial fibrosis and tissue characterization with PET’s ability to detect inflammation and metabolic activity can lead to a more accurate diagnosis and understanding of treatment-related cardiac damage [103]. These combined imaging approaches could provide an even more precise evaluation of cardiotoxicity, guiding treatment decisions and improving long-term outcomes.

## 7. Conclusions

In summary, the review underscores the essential role of advanced cardiac imaging modalities in the management of cancer patients with extracardiac tumors. The integration of techniques such as echocardiography, cardiac CT, and CMR has been instrumental in the early detection of cardiotoxicity, enabling timely intervention and improving patient outcomes. These imaging tools provide detailed insights into both the structural and functional aspects of cardiac health, which are critical in monitoring the cardiovascular effects of cancer therapies [3].

The integration of multimodal imaging in cardio-oncology requires a tailored approach to ensure optimal patient care. Echocardiography should be considered the first-line imaging modality for regular follow-up due to its accessibility and ability to detect early cardiac dysfunction, such as changes in GLS or myocardial work. Cardiac MRI is recommended for high-risk patients or those with significant findings, providing detailed tissue characterization, while cardiac CT plays a valuable role in assessing coronary artery disease in patients with a history of thoracic radiotherapy or those treated with chemotherapeutic agents that increase the risk of coronary artery disease.

In clinical practice, imaging strategies should be adapted based on the patient’s risk profile and the resources available in each medical setting. For resource-limited environments, prioritizing echocardiography and reserving advanced techniques for selected cases may optimize the use of available tools. These general recommendations aim to assist clinicians in planning follow-up protocols and improving outcomes for patients undergoing anticancer treatment.

The importance of collaboration between cardiologists and oncologists cannot be overstated. A multidisciplinary approach ensures that cancer patients receive optimal care, balancing the need for effective oncologic treatment with the prevention of cardiac complications. By working together, these specialists can develop individualized treatment plans that prioritize both cancer remission and cardiovascular health, ultimately enhancing the quality of life and survival of cancer patients [104]. As cardiac imaging continues to evolve, it will remain a cornerstone of comprehensive cancer care, ensuring that both oncologic and cardiovascular outcomes are optimized.

## Data Availability

The original data presented in the study can be found in the bibliographic references.
